# Physical Activity, Eating Habits and Mental Health during COVID-19 Lockdown Period in Serbian Adolescents

**DOI:** 10.3390/healthcare10050834

**Published:** 2022-04-30

**Authors:** Višnja Đorđić, Milan Cvetković, Boris Popović, Danilo Radanović, Milica Lazić, Biljana Cvetković, Slobodan Andrašić, Svetlana Buišić, Miroslav Marković

**Affiliations:** 1 Faculty of Sport and Physical Education, University of Novi Sad, 21101 Novi Sad, Serbia; djordjicvisnja@gmail.com (V.Đ.); borispopovic0803@gmail.com (B.P.); daniloradanovic@uns.ac.rs (D.R.); 2 Department of Psychology, Faculty of Philosophy, University of Novi Sad, 21101 Novi Sad, Serbia; milica.lazic@ff.uns.ac.rs; 3 Institute of Food Technology in Novi Sad, University of Novi Sad, 21101 Novi Sad, Serbia; biljana.cvetkovic@fins.uns.ac.rs; 4 Faculty of Economics, University of Novi Sad, 24000 Subotica, Serbia; slobodan.andrasic@ef.uns.ac.rs; 5 Faculty of Education in Sombor, University of Novi Sad, 21101 Novi Sad, Serbia; svetlana.buisic@pef.uns.ac.rs; 6 Institute for Improvement of Education, 11000 Belgrade, Serbia; miroslav.markovic@zuov.gov.rs

**Keywords:** body movement, nutrition, well-being, isolation, adolescents

## Abstract

The COVID-19 pandemic has impacted almost every aspect of life, especially daily physical activity and healthy eating habits but also mental health. Our study aimed to examine the relationship between the physical activity level, eating habits and mental health of Serbian adolescents during the COVID-19 pandemic. A total of 3506 students from the territory of the Republic of Serbia participated in this study. IPAQ-short version and HBSC-FFQ were used to assess physical activity level and eating habits, along with self-rated health. Moderate correlations were identified between physical activity, eating habits and mental health, along with average physical activity, very high life satisfaction (β = 0.177, *p* < 0.01) and very low emotional distress (β = −0.150, *p* < 0.01). A significant predictor of mental health was the frequency of breakfast on weekdays (β = 0.167, *p* < 0.01 for life satisfaction and β = −0.153, *p* < 0.01 for emotional distress), but not on weekends. Since the pandemic’s course is uncertain, the focus should be on maintaining good physical activity, nutrition and well-being.

## 1. Introduction

The COVID-19 pandemic is present in almost all countries of the world and thus leading to change in human everyday life. Restrictive measures introduced to prevent the spread of the pandemic have consequently encouraged a sedentary lifestyle in children and adolescents, neglecting daily recommendations for physical activity [1]. Beside mentioned daily consequences, the pandemic could show longer-term effects on children’s health, especially on childhood obesity [2]. However, apart from the obvious problem of preserving physical health, the COVID-19 pandemic has also caused mental health problems in children and adolescents [3]. There have been serious concerns raised regarding the mental health of children and adolescents during the pandemic [4,5].

Having in mind that insufficient physical activity is often associated with an imbalance in a person’s psychological well-being and various psychological problems [6], lack of access to exercise and physical activity could be one of the main reasons for such conditions. COVID-19 restrictions may have considerable negative consequences for the life satisfaction and emotional well-being of adolescents. This is why major social restrictions and closed schools may have reduced experiences that can offer self-realization and overall affirmation. Additionally, imposed social distancing has caused isolation and loneliness, which are risk factors for mental health [7]. Frustration could be frequent in adolescents, with events and encounters that they have not experienced. The serious questions need to be considered especially after natural disasters, because problems such as worsening school performance and increased agitation and aggression may arise or become even more pronounced [8]. In addition to physical activity, the pandemic and quarantine may compromise maintaining a healthy and varied diet [9]. However, there is an inverse relationship, showing that negative psychological responses to COVID-19 could increase the risk of developing dysfunctional eating behaviors [10,11].

Several studies have investigated the changes concerning physical activity, eating habits and mental health influenced by the COVID-19 pandemic in adolescents [3,12,13,14,15,16,17]. Zhang et al. [13] found negative effects of social isolation on children’s physical activity and mood. Moreover, the authors showed that the decrease in physical activity had a negative influence on mood during the COVID-19 pandemic. In line with this, a recent study among Greek adolescents revealed significant relationships between physical activity, sedentariness, eating behavior and well-being [12]. Similarly, Chi et al. [17] found that physical activity and healthy eating behavior are the best predictors for high well-being among adolescents.

The COVID-19 pandemic has changed daily habits in people of all ages. Given the fact that daily physical activity and healthy eating habits are the most important predictors of mental health, it was of great interest to investigate these relationships in adolescent students in a state of pandemic. Therefore, the aim of this study was to examine the relationship between the level of physical activity, eating habits and mental health of Serbian adolescents during the COVID-19 pandemic. We hypothesized that physical activity and eating habits will have significant impact on mental health among adolescents during the COVID-19 pandemic.

## 2. Materials and Methods

### 2.1. Participants

A total of 3506 students from the territory of the Republic of Serbia participated in this study. These are students of the fifth (30.4%) and seventh grades (33.2%) of elementary schools, as well as the first grade (36.4%) of high schools. Table 1 shows a detailed description of the sample in this study. The sample is not representative of the population of Serbia: there is a slightly higher percentage of girls in the sample (59.1%), as well as of the inhabitants of Vojvodina (46.9%). Body height and weight were measured according to standardized anthropometric measurements in participating schools. Body weight was measured to the nearest 0.1 kg using portable digital scales (Omron BF214, Kyoto, Japan), while height was measured to the nearest 0.1 cm by portable stadiometer (Seca 213, Hamburg, Germany). Body mass index (BMI) was calculated as weight (kg) divided by height squared (m^2^). Participants voluntarily participated in the study, the research was anonymous, and participants could withdraw from the study whenever they wanted. The study procedures were approved by the Faculty of Sport and Physical Education Ethical Board (No. 22/2019).

### 2.2. Emotional Distress and Life Satisfaction

The outcome variables of mental health were emotional distress and life satisfaction. The Health Behaviour in School-aged Children (HBSC) survey was used to assess adolescent health behaviors and psychosocial determinants [18,19]. Three measures were used to determine emotional distress: feeling low, feeling irritable or bad-tempered, and feeling nervous. Participants rated how frequently they experienced these complaints, moving from “Seldom or never” to “every day”. Life satisfaction was evaluated with one question. Participants rated how generally they felt satisfied with their lives, and the response options ranged from 0 (the worst life possible) to 10 (the best life possible).

### 2.3. Physical Activity

For evaluating different physical activity levels, a shorter version of the International Physical Activity Questionnaire (IPAQ) was applied [20], where number of days and number of hours were extracted from the obtained data. The frequency of daily physical activity was categorized from 1 to 5, with 0 days of physical activity as category 1, 1–2 days as category 2, 3–4 days as category 3, 5–6 days as category 4 and 7 days of physical activity as category 5 [21]. In addition and in accordance with the WHO [22], the recommended physical activity was moderate-to-vigorous, at least 60 min a day, 5 days in a week. In our case, the final outcome was categorized as satisfactory or unsatisfactory activity if the individuals participated more or less than 300 min/week, respectively.

### 2.4. Eating Habits

We used the Health Behaviour in School-Aged Children Food-Frequency Questionnaire (HBSC-FFQ) to evaluate the eating habits among adolescents in the current study [23]. The original version of the questionnaire was previously validated and values for each item can be found in Vereecken et al. [24]. The answers in the questionnaire were given in a Likert-type scale (never, less than once a week, once a week but not every day, once a day every day, more than once every day).

### 2.5. Statistical Analysis

All analyses were conducted in the statistical package SPSS, version 24. Descriptive data are presented as means and standard deviation as well as percentages. All the variables satisfied the normality of their distributions according to Kolmogorov–Smirnov test (*p* > 0.05) as well as the homoskedasticity (Levene variance homogeneity test). Along with the correlation analysis, four separate hierarchical regression analyses were used to test the hypotheses about the effect of physical activity and eating habits on students’ mental health during the pandemic. One set of predictors consisted of indicators of eating habits, the other set of predictors consisted of indicators of physical activity, the criterion variables were life satisfaction and emotional distress, and in each regression analysis, we used gender and age as control variables. We interpreted the standardized regression coefficients (β) as effect size estimates using the following interpretation: weak ≥ 0.10, moderate ≥ 0.30, strong ≥ 0.50.

## 3. Results

Table 2 shows descriptive statistics as well as correlations between study variables. Two measures of mental health (life satisfaction and emotional distress), two measures of physical activity, as well as two measures related to the frequency of breakfast (during working days and weekends), achieved a moderate correlation as expected. The correlation between measures related to the frequency of consumption of different groceries ranged from significance to moderate. All other variables’ correlations were low or negligible. On average, participants reported very high life satisfaction and very low emotional distress. The frequency of physical activity was around the theoretical average. Finally, participants achieved a BMI of 20.23 on average, and the score on Coca-Cola consumption is slightly lower than the theoretical average, while for all other groceries the score is slightly higher.

### 3.1. Contributions of Physical Activity to Life Satisfaction and Emotional Distress

In order to test whether physical activity makes a significant contribution to the explanation of mental health during a pandemic, two separate hierarchical regression analyses were conducted (for life satisfaction and emotional distress as criterion variables) (Table 3). The models are statistically significant in the first and second blocks, in the case of both criterion variables. After controlling for gender and age, the frequency of physical activity makes a significant contribution to explaining both life satisfaction (β = 0.144, *p* < 0.01) and emotional distress (β = −0.110, *p* < 0.01) (weak effect size), while the length of intense physical activity is not a significant predictor of either criterion. As the frequency of physical activity increases, life satisfaction also increases and emotional distress decreases in students during a pandemic.

### 3.2. Contributions of Eating Habits to Life Satisfaction and Emotional Distress

In order to examine the contribution of eating habits indicators to mental health during a pandemic, two separate hierarchical regression analyses were applied (Table 4). When it comes to both criterion variables, the model is statistically significant in both blocks. The body mass index negatively predicts life satisfaction (β = −0.052, *p* < 0.01), predicting emotional distress (β = 0.064, *p* < 0.01), although these are negligible effect sizes. A statistically significant predictor of mental health indicators was the frequency of breakfast on weekdays (β = 0.152, *p* < 0.01 for life satisfaction, and β = −0.122, *p* < 0.01 for emotional distress) (weak effect size), but not on weekends. While the frequency of Coca-Cola consumption was not a statistically significant predictor of mental health, fruit consumption positively predicted life satisfaction (β = 0.102, *p* < 0.01) and negatively predicted emotional distress (β = −0.070, *p* < 0.01), while the converse was predicted after consuming sweets (β = −0.047, *p* < 0.01 for life satisfaction and β = 0.108, *p* < 0.01 for emotional distress). The frequency of vegetable consumption was positively related to emotional distress (β = 0.076, *p* < 0.01), although with a negligible effect size.

## 4. Discussion

The aim of this study was to examine the relationship between the level of physical activity, eating habits and mental health of Serbian adolescents during the COVID-19 pandemic. The main findings of this study were that increased frequency of physical activity decreases emotional stress and increases life satisfaction in adolescents during COVID-19. Regarding eating habits, there was a slightly higher consumption of fruits, vegetables and sweets than the average. In addition, breakfast was a significant predictor of mental health, and eating fruits positively predicts life satisfaction and negatively predicts emotional distress, while sweets predict the opposite. Therefore, our hypothesis that physical activity and eating habits would be a significant predictor of mental health during COVID-19 pandemic could be accepted.

Physical activity can be defined as activity done with a large amount of effort, resulting in a substantially higher heart rate and energy expenditure [25,26]. However, due to the novel coronavirus disease 2019 (COVID-19), physical activity assessment is currently concerning [27]. Since our study has identified significant effects of physical activity on life satisfaction (β = 0.144, *p* < 0.01) and emotional distress (β = −0.110, *p* < 0.01), our results are in accordance with Greek adolescents who managed to maintain active lifestyle and satisfactory well-being [12]. The obtained results can be explained with several mechanisms. Changed circumstances can cut off some behavioral patterns through habit incoherence, which can lead to new health habit formation [28]. In regard to the absence of school obligations, adolescents were spending more time in activities that they usually do not have time for (family time, housework, learning new activities, quality sleep time, etc.) [29]. In order for the adolescents to be even more creative, some of them were also conducting online workouts, cycling and virtual dance classes [30,31]. Dunton et al. [31] also reported that 71% of adolescents were conducting activities with their parents, compared to 17% with their friends. In this regard, positive relation of physical activity with better well-being have also been reported elsewhere [32].Contrary to our results, a higher prevalence of physical inactivity was noted in Latin countries [33,34], and in that regard, their case was already an initially higher level of physical inactivity. Therefore, it is crucial to take into account differences in culture and tradition in order to completely understand the results.

A significant predictor of well-being is healthy eating [17]. It is also well-documented that an unhealthy way of eating can have a negative effect on mental state and cognitive functioning [35]. We have identified higher consumption of fruits, vegetables and sweets, which is similar to the results obtained by some other studies, explained further in the following. In Italy, Pietrobelli et al. [36] reported increased fruit intake, with no changes in vegetables, while sweets and sugary drink intake increased significantly during the COVID-19 pandemic, just like in the Palestinian study by Allabadi et al. [37]. In Cyprus, Konstantinou et al. [38] reported increased consumption of food items containing sugar, and if we add movement restrictions, the lockdown imposed an overall negative effect in adiposity in adolescents [36]. Hence, unhealthy food consuming has been on the rise [39]. These results should be taken with caution, based on differences in populations’ economic statuses, habits of eating and overall conducting of physical activities [40]. In addition, there should be also mention made that during lockdowns, people had limited opening hours to visit stores. However, on the other hand, people were also encouraged to prepare home-cooked meals, rather than ordering from restaurants [41]. With certainty, we cannot say that it was a healthier choice, but it should be taken into consideration that home-made quality over quantity of meal plays a big role. Likewise, based on the background and educational level of individuals, the whole concept of healthy eating varies [42].

During the COVID-19 pandemic, people had a tendency to be anxious and stressed regarding a shortage of food in the future. This fear resulted in purchasing packaged and non-perishable food instead of fresh food, which impacted the quality and led to gaining weight and increased intake of oxidants [43,44]. In addition, reduced energy expenditure also contributed to the development of obesity [45]. Since we found that frequency of physical activity makes significant contributions to explaining both life satisfaction and emotional distress, exercise is the most useful prevention tool for traumatic stress [46], as well as for weight control and overall immune system health [45].

This study have a few important limitations. The first one is that authors did not conducted any additional evaluation test concerning the type of conducted physical activities during both lockdowns. Since these adolescents stayed home all the time during isolations, sedentary behavior and sleep duration were not measured as well, while the third limitation applies to the actual physical activity during online classes. Therefore, some results should be taken with precaution. Another limitation is certainly the inadequate selection of measurement instruments, especially for physical activity. Moreover, there are no participants from some parts of Serbia. However, this was only a small proportion, which could not have significant impact on the results.

## 5. Conclusions

This study provides an overview of physical activity, eating habits and mental health during the COVID-19 lockdown period in Serbian adolescents and their moderate correlation. The results of the current study show significant impact of physical activity and eating habits on emotional distress and life satisfaction in adolescents during COVID-19. Since the course of the pandemic is uncertain at the moment, these results could provide information for maintaining healthy lifestyles, preserving good eating habits and well as managing stress in adolescents. The focus should be on maintaining productivity, nutrition and well-being, as well as reducing negative consequences.

## Figures and Tables

**Table 1:** Sample Description.

Variable	Percentage
Grade/Age	
5th grade/12.4 ± 0.8 (elementary school)	30.4%
7th grade/14.1 ± 0.6 (elementary school)	33.2%
1st grade/16.2 ± 1.3 (high school)	36.4%
Gender	
Male	40.9%
Female	59.1%
Region	
Belgrade	13.2%
Southern and Eastern Serbia	15.9%
Šumadija and Western Serbia	23.9%
Vojvodina	46.9%

**Table 2:** Descriptive Statistics and Correlations.

	1.	2.	3.	4.	5.	6.	7.	8.	9.	10.	11.
Life satisfaction	-										
Emotional distress	−0.43 **	-									
Physical activity	0.18 **	−0.16 **	-								
Intense physical activity	0.09 **	−0.08 **	0.43 **	-							
Body mass index	−0.05 **	0.07 **	−0.14 **	−0.04 *	-						
Breakfast (working days)	0.19 **	−0.18 **	0.11 **	−0.03	−0.10 **	-					
Breakfast (weekend)	0.13 **	−0.15 **	0.13 **	0.07 **	−0.15 **	0.56 **	-				
FoC Coca-Cola	−0.02	0.09 **	−0.07 **	−0.09 **	0.04 *	−0.09 **	−0.09 **	-			
FoC Fruit	0.10 **	0.00	0.22 **	0.16 **	−0.06 *	0.10 **	0.10 *	0.02	-		
FoC Vegetable	0.06 **	0.07 **	0.15 **	0.12 **	−0.03	0.11 **	0.10 **	0.04 *	0.68 **	-	
FoC Sweets	−0.03	0.14 **	−0.04 *	−0.07 **	−0.06 **	0.03	0.06 **	0.36 **	0.27 **	0.29 **	-
Mean	8.27	1.00	4.96	3.39	20.23	4.66	1.76	1.66	3.83	3.77	3.12
Standard deviation	1.67	0.93	2.02	1.66	3.91	0.82	0.55	1.66	1.71	1.65	1.70

** *p* < 0.01; * *p* < 0.05.

**Table 3:** Multiple Regression Analysis: Contribution of Physical Activity to Life Satisfaction and Emotional Distress.

	Life Satisfaction					Emotional Distress		
	R	B	S.E.	β	R	B	S.E.	Β
Block 1	0.184 **				0.283 **			
Gender _(female = 1)_		−0.061	0.057	−0.018		0.312	0.031	0.165 **
Age1 _(7th grade = 1)_		−0.387	0.070	−0.109 **		0.253	0.038	0.129 **
Age2 _(1st grade of high school = 1)_		−0.745	0.069	−0.215 **		0.483	0.037	0.251 **
Block 2	0.243 **				0.308 **			
Gender _(female = 1)_		−0.022	0.057	−0.006		0.295	0.031	0.156 **
Age1 _(7th grade = 1)_		−0.345	0.069	−0.097 **		0.235	0.038	0.120 **
Age2 _(1st grade of high school = 1)_		−0.670	0.069	−0.193 **		0.451	0.038	0.234 **
Physical activity		0.119	0.015	0.144 **		−0.051	0.008	−0.110 **
Intense physical activity		0.032	0.019	0.031		−0.015	0.010	−0.026

** *p* < 0.01.

**Table 4:** Multiple Regression Analysis: Contribution of Eating Habits to Life Satisfaction and Emotional Distress.

	Life Satisfaction				Emotional Distress			
	R	B	S.E.	β	R	B	S.E.	Β
Block 1	0.192 **				0.286 **			
Gender _(female = 1)_		−0.078	0.062	−0.023		0.315	0.034	0.167 **
Age1 _(7th grade = 1)_		−0.373	0.078	−0.106 **		0.247	0.043	0.125 **
Age2 _(1st grade of high school = 1)_		−0.766	0.075	−0.226 **		0.486	0.041	0.256 **
Block 2	0.259 **				0.352 **			
Gender _(female = 1)_		−0.077	0.062	−0.023		0.303	0.034	0.161 **
Age1 _(7th grade = 1)_		−0.356	0.077	−0.101 **		0.223	0.042	0.113 **
Age2 _(1st grade of high school = 1)_		−0.664	0.076	−0.196 **		0.423	0.041	0.223 **
Body mass index		−0.024	0.008	−0.052 **		0.016	0.004	0.064 **
Breakfast (working days)		0.202	0.034	0.152 **		−0.091	0.018	−0.122 **
Breakfast (weekend)		−0.052	0.077	−0.017		−0.065	0.041	−0.039
*Frequency of consumption*								
Coca-Cola		0.010	0.020	0.010		0.016	0.011	0.029
Fruit		0.100	0.024	0.102 **		−0.038	0.013	−0.070 **
Vegetable		−0.011	0.020	−0.011		0.043	0.014	0.076 **
Sweets		−0.046	0.020	−0.047 **		0.059	0.011	0.108 **

** *p* < 0.01.

## Data Availability

All data are available from the corresponding author on reasonable request.
